# A Rare Case of Small Cell Lung Cancer With an Epidermal Growth Factor Receptor Mutation and Its Response to Osimertinib

**DOI:** 10.7759/cureus.15136

**Published:** 2021-05-20

**Authors:** Sakil Bhuiyan, Raheel S Siddiqui, Milana Zirkiyeva, Mariam Agladze, Tayyaba Bashir

**Affiliations:** 1 Internal Medicine, Icahn School of Medicine at Mount Sinai, Queens Hospital Center, New York City, USA; 2 Hematology and Medical Oncology, Icahn School of Medicine at Mount Sinai, Queens Hospital Center, New York City, USA

**Keywords:** tki, egfr, sclc, nsclc, osimertinib

## Abstract

Small cell lung cancer (SCLC) accounts for less than 15% of the cases of lung cancer. Epidermal growth factor receptor (EGFR) mutations are rarely reported in association with SCLC. EGFR tyrosine kinase inhibitors (TKI) are approved as the first-line therapy for metastatic non-small cell lung cancer (NSCLC). The clinical effect of EGFR mutations and its response to osimertinib are unknown in SCLC. We report a case of EGFR-positive metastatic SCLC in a 63-year-old female who was treated with the third-generation TKI, osimertinib.

## Introduction

Lung cancer is the leading cause of cancer-related mortality in the world [1]. Lung cancers are broadly classified into small cell lung cancer (SCLC) and non-small cell lung cancer (NSCLC) based on microscopic features of the tumor cells. Epidermal growth factor receptor (EGFR) mutations are present in one-third of the patients with NSCLC, with a higher prevalence in females and non-smokers and around two-third of the patients having adenocarcinoma on histology [2]. EGFR tyrosine kinase inhibitors (TKI) are the first-line of therapy for metastatic EGFR-receptor-positive NSCLC due to better clinical outcome and lesser toxicity profile as compared to standard chemotherapy [3-5]. 

SCLC is a neuroendocrine tumor that accounts for less than 15% cases of lung cancer [6]. SCLC has been shown to respond to platinum-based chemotherapy and immunotherapy. Often, a combination therapy has been the mainstay of treatment. EGFR mutations are rarely reported with SCLC in literature [7-9]. We present a case of a 63-year-old female with SCLC associated with EGFR mutation that was treated with the third-generation EGFR-TKI, Osimertinib.

This article has been submitted as an abstract at https://afmr.org/Eastern/2021/24.cgi.

## Case presentation

**Figure 1:**
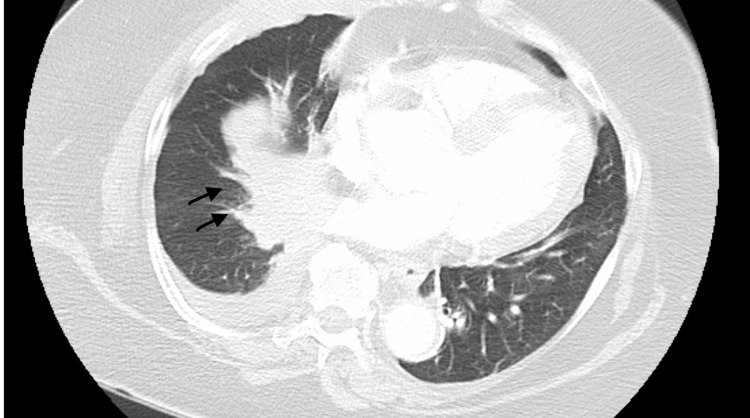
Initial CT chest with contrast showed hilar involvement of mass.

A 63-year-old woman, never smoker, presented to the hospital with a complaint of cough and blood in sputum for one month. Cachexia was noted on physical examination. Initial CT chest showed two right-lower lobe lung nodules, with the largest nodule measuring 2.1 cm, as well as pleural-based nodule and enlarged mediastinal lymph nodes (Figure 1).

**Figure 2:**
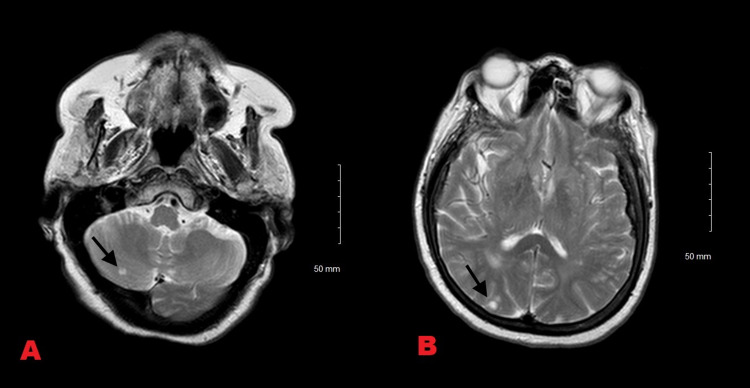
A. MRI brain showing a nodule in the right cerebellar hemisphere (arrow). B. Showing another nodule in the right occipital lobe.

CT abdomen pelvis revealed a pancreatic mass. MRI brain showed several nodular densities suggestive of metastatic disease despite absence of symptoms (Figures 2A, 2B).

**Figure 3:**
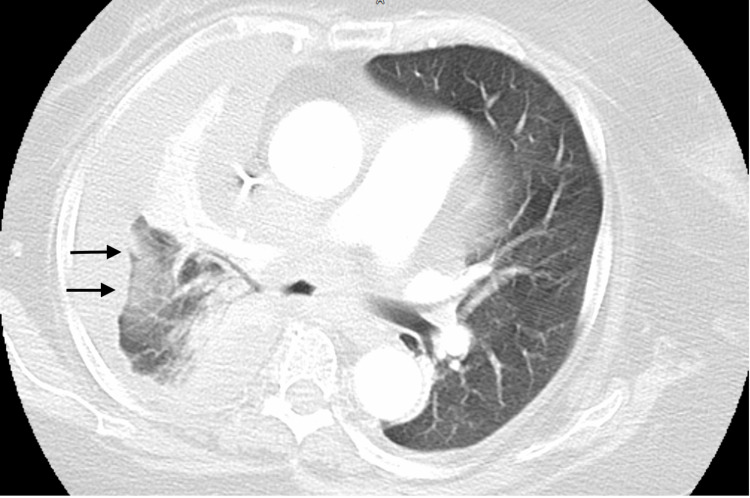
Follow up CT chest three months later showed compressive atelectasis of the right lung (arrows).

The patient eventually underwent a bronchoscopy, where endobronchial biopsies were obtained from the right-lower lobe lesions. Bronchial brushings were positive for malignant cells. The initial biopsy results revealed clusters of atypical cells with severe crushing effects consistent with SCLS. Immunohistochemistry revealed SCLC with EGFR positive for Exon 21 2573T>G L858R mutation as well as a high proliferative index (ki-67, 80%), and positive TTF-1 and synaptophysin. Tumor cells were negative for chromogranin and p63. The patient was classified as a Stage IV SCLC. Given the mildly symptomatic disease, the patient was started on targeted therapy with osimertinib with close monitoring of the pulmonary status. The patient completed about two months of osimertinib when her clinical status worsened and she required hospitalization after developing pneumonia and new pleural effusion. Follow-up CT chest revealed new mediastinal lymph nodes with pneumonia and worsening atelectasis (Figure 3).

The patient was subsequently treated for pneumonia with antibiotics without any improvement in symptoms. A decision was made to stop TKI therapy and start palliative chemotherapy with carboplatin and etoposide. The patient’s medical condition deteriorated rapidly and she opted for home hospice care.

## Discussion

The standard of care treatment for extensive stage (ES) SCLC is a combination of anti-PD 1 monoclonal antibody with etoposide-platinum chemotherapy. Alemtuzumab in combination with etoposide and carboplatin for patients with ES-SCLC showed an overall survival of 12.3 months vs 10.3 in months in patients with etoposide and carboplatin alone [10]. Similarly, a randomized clinical trial showed an overall survival of 13 months in durvalumab plus platinum-etoposide group versus 10.3 months in the etoposide-platinum alone group [11]. Although SCLC and NSCLC are histologically distinct entities with completely different treatment options, a study of 177 cases of SCLC has shown that 17 cases had components of NSCLC on histology and seven of these patients received a diagnosis of NSCLC initially [12]. Another common cause of concern in NSCLC is acquired mutations to TKI by histological conversion to SCLC. One study reported that out of 37 patients who developed resistance to TKI, five (14%) had histological conversion to SCLC [13]. Okamato et al. reported a case of exon 19 deletion positive EGFR mutation in a patient with metastatic SCLC who received treatment with TKI gefitinib and showed regression of both primary lung lesion and metastatic liver lesion [7]. 

The third-generation TKI, osimertinib, is approved as the first-line therapy for metastatic NSCLC with EGFR exon 19 deletion or exon 21 L858R mutation. Treatment with osimertinib showed a median progression-free survival of 18.9 months compared to 10.2 months when treated with first-generation EGFR TKIs (gefitinib or erlotinib) [14]. Our patient had metastatic SCLC with EGFR L858R mutation and a decision was made to attempt osimertinib as an initial therapy after the discussion of benefits and risks of standard platinum-based chemotherapy and immunotherapy.

In our patient, treatment with osimertinib did not show any meaningful improvement and the disease continued to progress. However, in our patient, TKI was not used in combination with cytotoxic chemotherapy. Studies have shown that after NSCLC transformation to SCLC, the SCLC responds to standard chemotherapy with platinum and etoposide [15]. The emergence of EGFR mutation in SCLC is relatively recent and studies have shown conflicting results of the efficacy of TKIs. This underscores the importance of further studies to see if TKIs, either alone or in combination with standard chemotherapy, show any better outcomes than the standard of care.

## Conclusions

TKI shows a good clinical response and is approved as the first-line therapy for metastatic EGFR mutation-positive NSCLC. The detection of EGFR mutation in SCLC underlines the importance of further studies to evaluate the efficacy and safety of TKIs, alone or in combination with standard of care, for the treatment SCLC.
